# Extradyadic Behaviors and Gender: How Do They Relate With Sexual Desire, Relationship Quality, and Attractiveness

**DOI:** 10.3389/fpsyg.2019.02554

**Published:** 2020-03-03

**Authors:** Joana Arantes, Fátima Barros, Helena M. Oliveira

**Affiliations:** School of Psychology, University of Minho, Braga, Portugal

**Keywords:** extradyadic behaviors, sexual desire, relationship quality, attractiveness, gender

## Abstract

Recent years have seen an increasing number of studies on relationship extradyadic behaviors (Pinto and Arantes, 2016; Pazhoohi et al., 2017; Silva et al., 2017; Fisher, 2018). However, much is still to learn about the impact of these extradyadic behaviors on subsequent relationships that an individual may have. Our main goal was to study the association between past extradyadic behaviors – inflicted and suffered – and current relationship quality, sexual desire and attractiveness. Specifically, we aimed to: (i) Understand if past extradyadic behaviors are related to current relationship quality, sexual desire, and self-perceived and partner’s attractiveness; (ii) Identify possible gender differences in these variables. For that, 364 participants (251 females and 113 males) were recruited through personal and institutional e-mails, online social networks (e.g., Facebook), and the website of the Evolutionary Psychology Group from the University of Minho. All participants completed a demographic and relationship questionnaire, followed by questions related to extradyadic behaviors and self-perceived attractiveness, the Perceived Relationship Quality Components (PRQC) Inventory, the Sex Drive Scale (SDQ), and the Importance of Partner’s Physical Attractiveness Scale (IPPAS). For those currently involved in a relationship, results suggested that extradyadic behaviors (both suffered or inflicted) are linked with current low relationship quality and high sexual desire in the present. In addition, individuals who perceived themselves as being more attractive tended to have a higher sexual desire and higher relationship quality. Overall, men reported higher levels of extradyadic behaviors and sexual desire, gave more importance to physical attractiveness, and perceived their current relationship as having less quality than women. These results add to the literature by focusing on different variables that play an important role in romantic relationships, and have important implications.

## Introduction

The experience of extradyadic behaviors without the primary partner’s prior consent can be the most painful occurrence for someone who is in an intimate relationship (Luo et al., 2010; Shrout and Weigel, 2018). Specifically, these extradyadic behaviors can have serious consequences, such as low self-esteem, mental problems, loss of trust, decreased personal and sexual confidence, rage, and guilt, and in many cases leads to spousal battering and divorce (Cano and O’Leary, 2000; Shackelford, 2001; Fisher et al., 2008; Russell et al., 2013). Studies have shown that these negative emotions can be found in both married and unmarried couples experiencing extradyadic behaviors (Mapfumo, 2016; Fincham and May, 2017; Shrout and Weigel, 2018). Extradyadic behaviors also affect other family members, especially children (Wilson et al., 2011). For example, parents that are not able to cope successfully with extradyadic behaviors are likely to expose their children to increase conflict (Blodgett-Salafia et al., 2013), and to trauma and grief like symptoms (Dean, 2011). In addition, extradyadic behaviors can lead to guilt, worry, fear, aggression, depression, and anxiety in children (Lusterman, 2005; Ablow et al., 2009; Negash and Morgan, 2015). Furthermore, the involvement in extradyadic behaviors are a major cause of seeking couples therapy and poses strong challenges for treatment (Gordon et al., 2004; Atkins et al., 2005, 2010; Marín et al., 2014). Our main goal was to investigate the relationship between past extradyadic behaviors, both inflicted and suffered, on current romantic relationships.

The majority of people have similar beliefs regarding extradyadic behaviors, expecting monogamy in marriage and overwhelmingly disapproving of extradyadic affairs (Prins et al., 1993; Buss and Shackelford, 1997; Treas and Giesen, 2000; Johnson et al., 2002; Rokach and Philiibert-Lignières, 2015). Sexual extradyadic behaviors tend to be considered more negative and hurtful than emotional behaviors (Rodrigues et al., 2016; Beltrán-Morillas et al., 2019). Although most of the studies are from the western cultures (Fisher, 2018; Norona et al., 2018), there are several studies that show similarities between cultures (e.g., Penn et al., 1997; Jankowiak et al., 2002; Nowak et al., 2014). For example, a cross-cultural study with a sample of 186 societies (e.g., South America, sub-Saharan Africa) found that in every culture, both males and females actively resort to mate-guarding tactics in order to try to control their mate’s extradyadic behaviors (Jankowiak et al., 2002). However, these beliefs do not always translate into consistent behaviors. In fact, studies have shown that approximately 22–25% of men and 15–20% of women report having sex with someone other than their spouse while been married (Allen et al., 2005; Mark et al., 2011). For example, in a study of Mark et al. (2011), 23.2% of men and 19.2% of women indicated that they had engaged in sexual interactions with someone other than their partner during their current relationship.

Is relationship satisfaction an important predictor of extradyadic behaviors? Research have showed that levels of general satisfaction with the relationship, sex, and the quality of love and affection are among the best predictors of extradyadic behaviors (Nowak et al., 2014; Fincham and May, 2017). Specifically, studies suggest that people engage in extradyadic behaviors because the quality of their primary relationship is low (Atkins et al., 2001; Scheeren et al., 2018). Glass and Wright (1985) found a negative correlation between relationship satisfaction and extradyadic behaviors – both emotional and sexual extradyadic behaviors –, being the strongest for relationship satisfaction and emotional extradyadic behaviors. Also, these authors found that those who are involved in both sexual and emotional extradyadic behaviors have lower levels of relationship satisfaction than those who are involved in an emotional-only affair, or a sexual-only affair. Extradyadic behaviors are associated with problems in primary relationship, especially for those with a stable secondary relationship (Fisher et al., 2009). DeMaris (2009) investigated some factors that might predict extradyadic affairs and found that relationship instability and poor communication were significant predictors. Rodrigues et al. (2016) showed that individuals in sexually monogamous relationships tended to report a higher predisposition for casual sex and to engage in extradyadic sex if they were in a low satisfaction relationship. Silva et al. (2017) found a positive correlation between perceived relationship quality and negative attitudes and perceptions of extradyadic behaviors, and that these correlations were stronger for men than for women. In addition, Negash et al. (2014) found that individuals who reported high relationship quality were more likely to end the relationship if they reported emotional or sexual extradyadic behaviors, suggesting that they had considerably more to lose when extradyadic behaviors occurred than those in low satisfaction relationships.

Buss and Shackelford (1997) found a link between relationship unhappiness and expectancies about partner’s extradyadic behaviors. Specifically, both men and women who feel generally unhappy with their primary relationship have a tendency to anticipate that their partners will have an extradyadic affair. Lower levels of relationship satisfaction seem to lead to extradyadic behaviors, and these extradyadic behaviors seems to lead to lower levels of relationship satisfaction as well (Buunk, 1980; Treas and Giesen, 2000; Atkins et al., 2001; Previti and Amato, 2004; Fincham and May, 2017).

It is important to note that not all studies found a significant correlation between relationship satisfaction and extradyadic behaviors (Blumstein and Schwartz, 1983; Spanier and Margolis, 1983; Glass and Wright, 1985). For example, Spanier and Margolis (1983), in a study with recently-separated and divorced individuals, concluded that the quality of sex between spouses was not directly related to extradyadic sex.

Sexual desire can be defined as specific sensations which move men and women to look out – or become receptive to – sexual experiences (Kaplan, 1979, 1995), or as a psychological state fundamental for initiating and maintaining human sexual behavior (Levine, 1988, 2002, 2003). Studies have shown that the tendency for sexual excitation is related to sexual desire (Janssen and Bancroft, 2007; Bancroft et al., 2009; Mark et al., 2011). Haseli et al. (2019) developed a systematic review about extradyadic behaviors and its associated factors, suggesting that features such as sexuality issues, including sexual desire, sexual interest, and sexual excitation affect the psychological processes to engage in extradyadic behaviors.

Treas and Giesen (2000) showed that the involvement in extradyadic behaviors were higher among men and women with stronger sexual interest levels (measured with a single item). However, McIntyre et al. (2015) found that stronger sexual desire predicted extradyadic behaviors only for individuals with low self-control. Fisher et al. (2009) stated that extradyadic behaviors may be related to interactions with a sexual partner, such as the partner’s hypoactive sexual desire. Fife et al. (2007) showed that extradyadic behaviors may occur when there is a high discrepancy in the sexual desire that both members of the couple experience.

Physical attractiveness is a predominant factor in sexual attraction, and research has shown that more attractive people are more prone to attract potential partners (Green et al., 1984). Extradyadic behavior seems to be an expression of mate choice continuing during a committed relationship, and mate choice largely reflects physical attraction (Nowak et al., 2014). For people involved in a romantic relationship, perceptions of the partner’s physical attractiveness is positively correlated with commitment, passion, intimacy, and satisfaction (Yela and Sangrador, 2001). In addition, McNulty et al. (2008) found that those involved in a relationship in which wives were more physically attractive than their husbands had higher levels of relationship quality. Some studies have shown that more physically attractive women are more likely to have extradyadic behaviors and to have more sexual partners (Singh, 1993; Dijkstra and Buunk, 2001; Hughes and Gallup, 2003; Streeter and McBurney, 2003) employing, among other measures, the observation of the waist-to- rip ratios (WHR) and women’s sexual behavior.

One of the most frequently-studied variables in extradyadic behaviors is gender (Symons, 1979; Lawson, 1988; Betzig, 1989; Feingold, 1990, 1992; Buss et al., 1992, 1999; DeSteno et al., 2002; Shackelford et al., 2002; Grammer et al., 2003; Sagarin et al., 2003; Kurzban and Weeden, 2005; Fisman et al., 2006; Kalantarova et al., 2010; Frederick and Fales, 2016; Pinto and Arantes, 2016). Several studies reported that men are involved in extradyadic behavior more than women (Greeley, 1991; Laumann et al., 1994; Wiederman, 1997), and that both genders engage in different types of extradyadic behavior. Specifically, men are more likely to have sexual affairs, whereas women are more likely to have emotional affairs (Glass and Wright, 1985; Atkins et al., 2001; Scheeren et al., 2018). Furthermore, men have a tendency to assess partner sexual extradyadic behaviors more negatively than emotional extradyadic behaviors (Tagler and Jeffers, 2013). However, studies also suggest that gender differences in extradyadic behaviors in younger generations are reducing in size, indicating that the rates of extradyadic behaviors are becoming increasingly similar among both men and women (Parker, 1997; Wiederman, 1997; Atkins et al., 2001; Pinto and Arantes, 2016).

## Our Study

As mentioned before, extradyadic behaviors can have a devastating impact on the couple. For example, the partner who is betrayed experiences frequently intense negative emotions such as depression, overwhelming powerless, abandonment, and victimization (Charny and Parnass, 1995; Gordon et al., 2004). Therefore, our main goal is to investigate the association between past extradyadic behaviors – inflicted and suffered –, and the experience of the current romantic relationships.

More specifically, we aim to (i) identify differences between men and women on sexual desire, attractiveness, extradyadic behaviors and relationship quality; (ii) understand if extradyadic behaviors are related to current sexual desire, attractiveness and relationship quality. Regarding the first specific aim, previous researchers have investigated gender differences on extradyadic behaviors (Wiederman, 1997), attractiveness (Lippa, 2007), and relationship quality (Silva et al., 2017). However, in the current study we aim to analyze whether males and females show different extradyadic behaviors with the current partner and/or a past partner, attribute different importance to the partner’s physical attractiveness and assess their own attractiveness differently, and score differently on a scale that evaluates the quality of their current romantic relationship. In what concerns the second specific aim, the emotional, cognitive, and behavioral responses that appear as a consequence of an affair may be comparable to those after a traumatic experience (Baucom et al., 2006). However, even though the short-term responses to an affair are the focus of several researchers (Pazhoohi et al., 2017), much is still to learn about the impact of extradyadic behaviors in subsequent relationships. Therefore, we will focus on the association between extradyadic behaviors (both past and current), sexual desire, attractiveness, and relationship quality.

We have four hypotheses:

Hypothesis 1 – Men have higher levels of extradyadic behaviors, higher levels of sexual desire, give more importance to physical attractiveness and perceive their current relationship as having less quality compared to women. This hypothesis is based on previous research that showed that women tend to have less extradyadic behaviors than men (Pinto and Arantes, 2016), and more negative attitudes and perceptions of extradyadic behaviors (Silva et al., 2017). Men are also shown to have higher sex drive (Baumeister et al., 2001) and attribute higher importance to partner’s attractiveness (Bailey et al., 1994). In addition, previous research has shown that females tend to have higher levels of overall perceived relationship quality compared to males (Silva et al., 2017).

Hypothesis 2 – Individuals that had betrayed a partner tend to have higher sexual desire, lower relationship quality, and to attribute higher importance to partner’s attractiveness. This hypothesis is based on previous studies suggesting that sexual desire may be related with extradyadic behaviors (Treas and Giesen, 2000), and that individuals that report more thoughtful decision-making processes regarding their romantic relationship also tend to be more satisfied with the relationship and have fewer extradyadic behaviors (Owen et al., 2013). In addition, prior research has shown that finding non-partners attractive is a predictor of extradyadic behaviors (Nowak et al., 2014).

Hypothesis 3 – Individuals that had been betrayed by a partner tend to have higher sexual desire, lower relationship quality, and to attribute lower importance to partner’s attractiveness. This hypothesis is based on prior research that showed that individuals that have been betrayed by a partner are more likely to have extradyadic behaviors as retribution (Scheeren et al., 2018).

Hypothesis 4 – Those who perceive themselves as being more attractive tend to have a higher relationship quality and higher sexual desire. Research has shown that higher body esteem is positively related to relationship quality (Erol and Orth, 2016) and to sexual desire (Seal et al., 2009). In addition, previous research has shown that for people involved in a romantic relationship, perceptions of the partner’s physical attractiveness is positively correlated with commitment, passion, intimacy, and satisfaction (Yela and Sangrador, 2001). In addition, studies have shown that more physically attractive women are more likely to have extradyadic behaviors (e.g., Dijkstra and Buunk, 2001).

## Methodology

### Participants

Our initial sample included 488 participants. After excluding incomplete questionnaires (*n* = 106) and all those from a nationality other than Portuguese (*n* = 18), our final sample had 364 participants, ranging from 18 to 62 years old (*M* = 26.10 years; *SD* = 7.77). Of those, 251 (68.96%) were female and 113 (31.04%) male. Overall, men (*M* = 28.59; *SD* = 9.16) were about 4 years older than women (*M* = 24.97; *SD* = 6.78), which is a statistically significant difference, *t*(17.30) = 3.76, *p* < 0.001. In terms of sexual orientation, 339 (93.13%) reported that they were heterosexual, 13 (3.57%) homosexual, and 12 (3.30%) bisexual. Most participants (*n* = 267; 73.35%) said they were currently involved in one intimate relationship. Of those participants involved in a relationship, the majority was involved in a dating relationship (*n* = 168; 62.92%), followed by marriage (*n* = 45), *de facto* relationship (*n* = 25), casual (*n* = 15), paramours (*n* = 9), and other (*n* = 5). Concerning relationship duration, 62 reported they had been in that relationship for less than one year, 71 from one to three years, 53 from three to five years, 50 from five to 10 years, 17 from 10 to 15 years, and 14 for more than 15 years.

### Measures

#### Demographic and Relationship Questions

Participants answered several demographic questions, including age (in years), gender (male or female), nationality, and sexual orientation (heterosexual, homosexual or bisexual). Participants were also asked if they were currently involved in one or more relationships (e.g., dating, partnership, marriage, casual, paramour). Those who responded affirmatively specified the duration of each relationship (less than 1 year, between 1 and 3 years, between 3 and 5 years, between 5 and 10 years, between 10 and 15 years or more than 15 years). This allowed a participant that had, for example, a partner and a paramour, to specify the duration of each relationship.

#### Extradyadic Behaviors’ Questions

Participants were asked about their history of extradyadic behaviors. Specifically, regarding their own extradyadic behaviors, they were asked: (i) if they had betrayed (or were betraying) their current partner with another person (yes or no); (ii) if they had betrayed other partners in the past (yes or no); and, if they answered affirmatively, (iii) with how many persons have they been with (while betraying a partner). Regarding participants’ partners extradyadic behaviors, participants were asked: (i) if they knew or believed they had been (or were being) betrayed by their current partner; (ii) if they had been betrayed by past partners; and, if they answered affirmatively, (iii) by how many partners.

#### Perceived Relationship Quality Components (PRQC) Inventory

Participants that were currently involved in a relationship completed the PRQC Inventory (Fletcher et al., 2000; Portuguese version translated and validated by Silva et al., 2017), in order to assess relationship quality. Participants were asked to rate their current partner and relationship on 18 items, divided into 6 perceived relationship quality components – relationship satisfaction, commitment, intimacy, trust, passion, and love. Each component was assessed by three items (e.g., “How satisfied are you with your relationship?”; “How committed are you to your relationship?”), each scoring on a 7-point Likert-type scale, with 1 = “not at all” and 7 = “extremely.” In addition to a total score computed as the arithmetic mean of all items, six mean scores were calculated, one for each component. Higher scores indicated greater perceived relationship quality. Fletcher et al. (2000) used confirmatory factor analysis to compare several models of how the components were related, and found evidence in favor of a more complex, higher-order model. This model included six first-order factors, representing the six components, and one second-order factor, representing the perceived relationship quality. According to Fletcher et al. (2000), this model produced an acceptable fit to the original data, with a CFI > 0.90 and an RMSEA = 0.08. The components also demonstrated good internal reliability (α = 0.88, Study 1; α = 0.85, Study 2; α = 0.97, Silva et al., 2017; α = 0.95, Current study).

#### Sex Drive Scale (SDQ)

The SDQ (Ostovich and Sabini, 2004) measures sex drive by four items, scored on a 7-point Likert-type scale, without requiring participants to have a romantic or sexual partner to be classified as high in sex drive (e.g., “How often do you experience sexual desire?”; “How often do you orgasm in the average month?”). Sex drive has been frequently operationalized as a sexual desire or as libido (Ostovich and Sabini, 2004; Lippa, 2006, 2009). A total score was aggregated by computing the arithmetic mean of the four items, with higher scores indicating greater sexual desire. According to Ostovich and Sabini (2004), the original scale consists of one factor that explains 62.8 and 66.3% of the variance in men’s and women’s scores, respectively.

#### Importance of Partner’s Physical Attractiveness Scale (IPPAS)

The IPPAS (Bailey et al., 1994) is a self-report measure that evaluates the importance of partner’s physical attractiveness. It includes 10 Likert-scaled items scored between 1 = “I strongly disagree” – and 7 = “I strongly agree.” The first six items are more general and include items such as “Looks aren’t that important to me,” and “It is easy to imagine becoming romantically involved with someone I initially felt was physically unattractive, as I grew to know his/her personality.” The last four items are specific to the current partner or, in case the participant is not involved in a romantic relationship, a hypothetical partner (Galperin and Haselton, 2010), and include items such as “I like my romantic partner to dress attractively, even if it requires some effort on his/her part,” and “If my partner became much less physically attractive, it would be difficult for me to stay with him/her.” Items one, two and four are inverted. Total scores were computed by calculating the arithmetic mean of the individual items, with higher scores indicating greater importance attributed to physical attractiveness.

#### Self-Perceived Attractiveness

Participants were asked to rate their own attractiveness, on a scale from 0 (“not attractive at all”) to 100 (“extremely attractive”).

### Procedure

Participants did not receive monetary compensation, and were recruited through personal and institutional e-mails, online social networks (e.g., Facebook), and the website of the Evolutionary Psychology Group from the University of Minho. Because the IPPAS and SDQ had not been previously used with a Portuguese-speaking population, we followed standard procedures for adapting scales in cross-cultural research (Geisinger, 1994). More specifically, after contacting the original authors, we translated the items individually into European Portuguese and discussed language adequacy for all items. The scales were then translated back to English by a bilingual researcher and compared with the original scales. No major discrepancies were noted. Finally, all items were discussed with other members of our lab for linguistic and theoretical adequacy.

Participants’ responses were recorded anonymously on an Internet webpage using Qualtrics software, Version 2013 of the Qualtrics Research Suite^1^. For all participants, demographic and relationship questions were presented first. Then, they answered to the SDQ, IPPAS and self-perceived attractiveness, in a counterbalanced order. Those participants that were currently involved in a relationship completed also the PRQC Inventory, and those participants that have ever been in a relationship (present or past) completed the extradyadic behaviors’ questions as well – both presented in a counterbalanced order, intermixed with the SDQ, IPPAS and self-perceived attractiveness.

### Data Analysis

The data were exported to an Excel spreadsheet. Analyses were conducted with Statistical Package for Social Sciences (SPSS; v. 21), and included exploratory factor analyses to validate the scales, correlations to evaluate the associations among variables, *t* tests and chi squares to examine gender differences, and Univariate analyses of variance (ANOVA) to compare whether males and females who had betrayed a partner and those who had not had significantly different scores in terms of the different variables in the study.

Confirmatory factor analyses were performed using AMOS software (v. 20). As recommended by Hu and Bentler (1999), we reported two indices of model fit, the Comparative Fit Index (CFI), and the Root Mean Square Error of Approximation (RMSEA). The exploratory factor analysis (EFA) was conducted on a random split-half sample of the data (*n* = 182) and the confirmatory factor analysis was conducted in the holdout sample (*n* = 182). A criterion of *p* < 0.05 was used for significance tests.

## Results

### SDQ and IPPAS Validation

Since the SDQ and the IPPAS had not been previously translated and validated to Portuguese, initially we investigated the psychometric properties of the translated scales. First, analyses of the scales’ sensitivity via frequency tables and distributions of the data showed that all items from both scales had good sensitivity (i.e., all response categories were represented in the sample).

Second, construct validity was assessed for both scales by exploratory and confirmatory factor analyses, after confirming the factorability of the data through the Bartlett sphericity test (*p* < 0.001 for both scales) and Kaiser-Meyer-Olkin test (SDQ:0.75; IPPAS:0.75). An exploratory factor analysis with Varimax rotation performed on the SDQ produced one factor, which accounted for 64.33% of the variance, and all the items had a loading ≥0.73. This result is consistent with the original instrument (Ostovich and Sabini, 2004). A confirmatory factor analysis showed a good fit of the one factor model to the SDQ (CFI > 0.90; RMSEA < 0.06), after error terms for items two and three were correlated. Regarding the IPPAS, an exploratory factor analysis extracted three factors. However, after examination of the scree plot and based on theoretical considerations, we forced the factor analysis to extract one factor. With exception of the last item (“I would be happy if my partner were more sexually attractive than I”), all items had a loading ≥0.41. Even though all items should have a loading >0.30 (Tabachnick and Fidell, 2001; Costelly and Osborne, 2005), after careful consideration regarding the content of the last item, we decided to retain this item in the Portuguese version. The one factor model accounted for 33.46% of the variance. A confirmatory factor analysis allowing for the errors of items 5 and 6, 7 and 8, and 8 and 9 to be correlated showed a good fit of the one factor model (CFI > 0.90; RMSEA < 0.06).

Third, the scales revealed good reliability (α = 0.80, SDQ; α = 0.74, IPPAS). Also, the Cronbach’s α did not increase considerably when any items were removed, confirming that all items should be retained for the scale. These results are consistent with those obtained with the original scales. More specifically, Ostovich and Sabini (2004) showed that the SDQ has good internal reliability (α = 0.79, men; α = 0.83, women). According to Bailey et al. (1994), the IPPAS has good internal reliability (α = 0.77, heterosexual women; α = 0.77, homosexual women; α = 0.70, heterosexual men; α = 0.75, homosexual men).

Fourth, distributions for individual variables were examined. Table 1 shows descriptive statistics for the full sample on both scales. Results are shown for the full sample, and separately for male and female participants. Regarding the SDQ, scores in our sample showed the same pattern as the original authors, with men reporting significantly higher sexual desire than women, *t*(243.39) = 13.45, *p* = 0.000. These results are also consistent with Haselton and Buss (2000) and Haselton (2003). Results also showed that male participants scored significantly higher than female participants on the IPPAS, which is consistent with Galperin and Haselton, *t*(362) = 7.03, *p* = 0.000.

**TABLE 1:** Descriptive statistics (*M* = Mean; *SD* = Standard Deviation) for the full sample, and for female and male participants separately.

	Full sample		Females		Males	
	*(n* = 364)		*(n* = 251)		*(n* = 113)	
			
	*M*	*SD*	*M*	*SD*	*M*	*SD*
SDQ	4.15	1.07	3.75	0.92	5.04	0.81
IPPAS	3.81	0.84	3.61	0.77	4.24	0.83

### Extradyadic Behaviors

Overall, for the extradyadic behaviors’ questions, 93 participants (25.55%) mentioned they have betrayed at least a partner in their lifetime, namely 44 men (38.94%) and 49 women (19.52%). More specifically, 90 participants (24.72%) indicated they had betrayed other partners in the past. Of those, 32 (35.56%) had one paramour, 36 (40.00%) had two paramours, 11 (12.22%) had three paramours, and 11 (12.22%) had four paramours. Men had betrayed significantly more in the past than women (Men: 37.17%; Women: 19.12%). This association was significant, χ^2^ (1, *N* = 364) = 13.63, *p* = 0.000. Conversely, a similar number of men and women (*n* = 161; 44.23%) indicated they had been betrayed by past partners, χ^2^ (1, *N* = 364) = 0.054, *p* = 0.82. Specifically, 110 (68.32%) indicated that they had been betrayed in the past by one partner, 50 (31.06%) by two partners, and one (0.62%) by three partners.

It is important to note that of the 267 participants (73.35% of the full sample) who were currently involved in an intimate relationship (e.g., dating, partnership, marriage, casual, paramour), only 7 (2.62%) said they had betrayed (or were betraying) their current partner with another person, and 11 (4.12%) reported that they knew (or believed) they had been (or were being) betrayed by their current partner. Therefore, in the analyses we present below, we combined all the participants that have ever betrayed a partner (i.e., a past or a current partner), and we combined all the participants that have ever been betrayed by a partner (i.e., a past or a current partner).

### Extradyadic Behaviors and Gender: How Do They Relate With Sexual Desire, Relationship Quality, and Attractiveness

**FIGURE 1:**
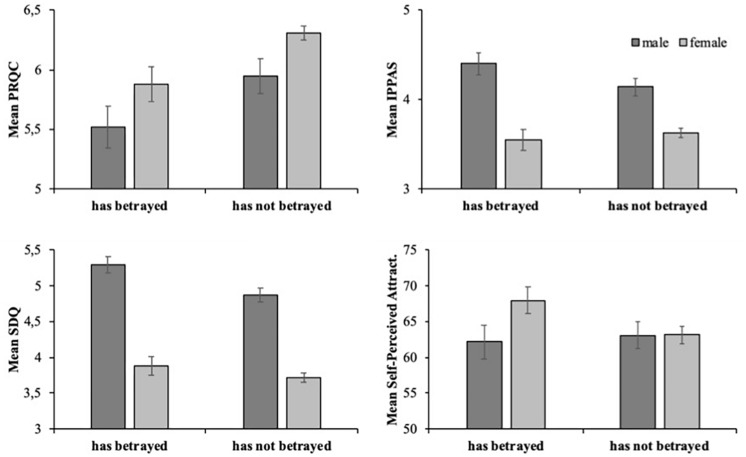
Average scores for PRQC **(upper left)**, IPPAS **(upper right)**, SDQ **(lower left)**, and Self-Perceived Attractiveness **(lower right)**, shown separately for males (dark gray) and females (light gray), and has betrayed and has not betrayed. Bars indicate + 1 SE.

Of particular interest was whether males and females who had betrayed a partner and those who had not had significantly different scores in terms of the variables listed in Table 2. Therefore, we divided participants into four groups depending on gender and whether they had or not betrayed a partner: male – had betrayed, *n* = 44; male – had not betrayed, *n* = 69; female – had betrayed, *n* = 49; and female – had not betrayed, *n* = 202. The average scores for the measures relating to relationship quality, sexual desire and attractiveness are shown in Figure 1. Univariate analyses of variance (ANOVA) with gender and had betrayed (yes/no) as factors were conducted for each measure.

**TABLE 2:** 2 × 2 contingency table with “Has betrayed/Has not betrayed” and “Has been betrayed/Has not been betrayed.”

	Has betrayed	Has not betrayed
	*(n* = 90)	*(n* = 274)
Has been betrayed (*n* = 161)	55 (59.14%)	108 (39.85%)
Has not been betrayed (*n* = 203)	38 (40.86%)	163 (60.15%)

These results showed that PRQC scores were higher overall for females (*M*_Men_ = 6.22, *SD*_Men_ = 0.75; *M*_Women_ = 5.75; *SD*_Women_ = 1.03), *F*(1,267) = 9.11, *p* = 0.033, partial η^2^ = 0.03. This result is consistent with Hypothesis 1. In addition, PRQC scores were higher for those males and females who had never betrayed (*M*_had not betrayed_ = 6.22, *SD*_had not betrayed_ = 0.77; *M*_had betrayed_ = 5.70, *SD*_had betrayed_ = 0.98), *F*(1,267) = 8.96, *p* = 0.000, partial η^2^ = 0.048. This result is consistent with Hypothesis 2. The interaction was not significant, *F*(1,267) = 0.00, *p* = 0.99, partial η^2^ = 0.000.

For SDQ, the average scores were higher for men (*M*_Men_ = 5.04, *SD*_Men_ = 0.81; *M*_Women_ = 3.75, *SD*_Women_ = 0.92), *F*(1,364) = 135.80, *p* = 0.000, partial η^2^ = 0.274. This result is consistent with Hypothesis 1. Furthermore, SDQ scores were also higher for those who had already betrayed (*M*_had not betrayed_ = 4.01, *SD*_had not betrayed_ = 1.03; *M*_had betrayed_ = 4.55, *SD*_had betrayed_ = 1.10), *F*(1,364) = 6.78, *p* = 0.010, partial η^2^ = 0.018. This result is consistent with Hypothesis 2. The interaction gender × betrayed was not significant, *F*(1,364) = 1.29, *p* = 0.257, partial η^2^ = 0.004.

For IPPAS, the main effect of gender was significant, where overall scores were higher for males (*M*_Men_ = 4.24, *SD*_Men_ = 0.83; *M*_Women_ = 3.61, *SD*_Women_ = 0.77), *F*(1,364) = 47.93, *p* = 0.000, partial η^2^ = 0.117. However, no significant differences were obtained between those males and females who had betrayed, and those who had never betrayed, *F*(1,364) = 0.77, *p* = 0.381, partial η^2^ = 0.002. The interaction gender x betrayed was also not significant, *F*(1,364) = 2.96, *p* = 0.086, partial η^2^ = 0.008. These results are consistent with Hypothesis 1 but inconsistent with Hypothesis 2. No significant differences were found for self-perceived attractiveness.

**FIGURE 2:**
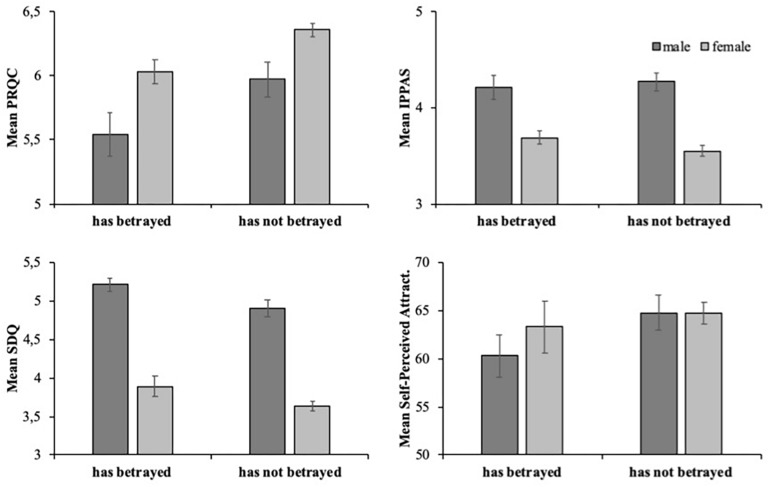
Average scores for PRQC **(upper left)**, IPPAS **(upper right)**, SDQ **(lower left)**, and Self-Perceived Attractiveness **(lower right)**, shown separately for males (dark gray) and females (light gray), and has been betrayed and has not been betrayed. Bars indicate + 1 SE.

We were also interested in whether participants who had been betrayed by a partner, and those who had not, scored differently in the variables of our study. Therefore, similar to the previous analysis, we divided participants into four groups depending on gender and whether they had or not been betrayed by a partner: male – had been betrayed, *n* = 51; male – had not been betrayed, *n* = 62; female – had been betrayed, *n* = 112; and female – had not been betrayed, *n* = 139. Figure 2 shows the average scores for the PRQC, IPPAS, SDQ and Self-Perceived Attractiveness. ANOVAs with gender and had betrayed were conducted for each measure.

For the PRQC, the scores were higher for females (*M*_females_ = 6.22, *SD*_females_ = 0.75; *M*_males_ = 5.75, *SD*_males_ = 1.03), *F*(1,267) = 16.02, *p* = 0.000, partial η^2^ = 0.057, a result consistent with Hypothesis 1. PRQC scores were higher for those males and females who had never been betrayed (*M*_not been betrayed_ = 6.25, *SD*_not been betrayed_ = 0.74; *M*_been betrayed_ = 5.87, *SD*_been betrayed_ = 0.96), *F*(1,267) = 11.81, *p* = 0.001, partial η2 = 0.043. This result is consistent with Hypothesis 3. The interaction was not significant, *F*(1,277) = 0.19, *p* = 0.662, partial η^2^ = 0.001.

For the SDQ, the average scores were higher for men (*M*_females_ = 3.75, *SD*_females_ = 0.92; *M*_males_ = 5.04, *SD*_males_ = 1.81), *F*(1,364) = 165.98, *p* = 0.000, partial η^2^ = 0.316, a result consistent with Hypothesis 1. SDQ scores were higher those males and females who had already been betrayed (*M*_not been betrayed_ = 4.02, *SD*_not been betrayed_ = 1.05; *M*_been betrayed_ = 4.30; *SD*_been betrayed_ = 1.07), *F*(1,364) = 8.22, *p* = 0.004, partial η^2^ = 0.022. The interaction was not significant, *F*(1,364) = 0.08, *p* = 0.779, partial η^2^ = 0.000. These results are inconsistent with Hypothesis 3.

For the IPPAS, the main effect of gender was significant, where overall scores were higher for males (*M*_females_ = 3.61, *SD*_females_ = 0.77; *M*_males_ = 4.24, *SD*_males_ = 0.83), *F*(1,364) = 47.30, *p* = 0.000, partial η^2^ = 0.116, a result consistent with Hypothesis 1. However, no significant differences were obtained between those males and females who had been betrayed, and those who had never been betrayed (*M*_not been betrayed_ = 3.77, *SD*_not been betrayed_ = 0.86; *M*_been betrayed_ = 3.85, *SD*_been betrayed_ = 0.80), *F*(1,364) = 0.22, *p* = 0.643, partial η^2^ = 0.001. The interaction gender x betrayed was also not significant, *F*(1,364) = 1.33, *p* = 0.249, partial η^2^ = 0.004. These results are inconsistent with Hypothesis 3. No significant differences were found for self-perceived attractiveness.

Table 2 shows the association between having betrayed and having been betrayed. The Phi coefficient confirmed that they were significantly related, φ = 0.17, *p* = 0.001, indicating that participants that have been betrayed are also more likely to have betrayed.

### Relationship Quality, Sexual Desire and Attractiveness: Correlation Analysis

We examined correlations among the relationship quality, sexual desire and attractiveness variables. Results are shown in Table 3.

**TABLE 3:** Correlations between age, perceived relationship quality (PRQC-Total), sexual desire (SDQ), importance given to partner’s attractiveness (IPPAS) and perceived self-attractiveness (*n* = 382).

	Age	PRQC	SDQ	IPPAS	Self-perceived attractiveness
**Age**					
PRQC	–0.20^∗∗^				
SDQ	0.15^∗∗^	–0.17^∗∗^			
IPPAS	0.12^∗^	–0.28^∗∗∗^	0.17^∗∗^		
Self-perceived attractiveness	–0.04	0.18^∗∗^	0.16^∗∗^	0.07	

*^∗∗∗^*p* < 0.001; ^∗∗^*p* < 0.01; ^∗^*p* < 0.05.*

Age was significantly negatively correlated with PRQC-Total, *r* = −0.20, *p* = 0.001, indicating that older participants perceived their relationship quality as been poorest than younger participants. Age was positively correlated with SDQ, *r* = 0.15, *p* = 0.004, showing that older participants reported higher levels of sexual desire than younger participants. Age was also positively correlated with IPPAS, *r* = 0.12, *p* = 0.019, indicating that older participants attribute more importance to the partner’s physical attractiveness than younger participants.

The responses to the PRQC were negatively correlated with SDQ, *r* = −0.17, *p* = 0.006, showing that those participants who perceived their relationship quality as being higher, tended to have less sexual desire. The PRQC was also negatively correlated with IPPAS, *r* = −0.28, *p* = 0.000, indicating that participants who perceived their relationship quality as being higher attributed less importance to the partner’s physical attractiveness. In addition, PRQC was positively correlated to self-perceived attractiveness, *r* = 0.18, *p* = 0.003, showing that participants who perceived their relationship quality as being higher tended to perceive themselves as being more attractive. This is consistent with Hypothesis 4.

Those who perceived themselves as being more attractive tended to have a higher sexual desire, *r* = 0.16, *p* = 0.002. This is consistent with Hypothesis 4. Finally, SDQ was positively correlated with IPPAS, indicating that participants who attributed more importance to the partner’s physical attractiveness reported higher levels of sexual desire, *r* = 0.17, *p* = 0.001.

Next we investigated whether males and females had significantly different correlations for the variables in Table 3. Results show that the positive correlation between perceived attractiveness and sexual desire was only obtained for women (*r* = 0.26, *p* = 0.000). Similarly, PRQC-Total was negatively correlated with IPPAS for women (*r* = −0.26, *p* = 0.000), but not for men. By contrast, the positive correlation between PRQC and self-perceived attractiveness was only obtained for men (*r* = 0.24, *p* = 0.030).

## Discussion

The primary goal of the present study was to examine the association between past extradyadic behaviors – both inflicted and suffered – on current romantic relationships. Results from our data showed that men have higher levels of extradyadic behaviors, higher levels of sexual desire, gave more importance to physical attractiveness and perceived their current relationship as having less quality compared to women. These results confirmed our first hypothesis. Findings are consistent with the existent literature (Ostovich and Sabini, 2004; Galperin and Haselton, 2010; Pinto and Arantes, 2016). For example, previous studies have showed that females tend to have fewer extradyadic behaviors (Pinto and Arantes, 2016). One possible explanation is that there are stereotypes and gender roles that have been internalized about women being good wifes (Bittman et al., 2003; Ellemers, 2018). Another possible explanation – based on an evolutionary perspective – is the greater maternal investment required for pregnancy and subsequent child care (Hill and Hill, 1990; Bjorklund and Shackelford, 1999). However, Wiederman and Hurd (1999) suggested that the differences in extradyadic behaviors obtained may be due to underreporting of extradyadic behaviors by women rather than real sex differences – due to the existent double sexual standard.

Those participants that have betrayed in the past are significantly more likely to perceive the quality of their current relationship as being lower and to have a higher sexual desire in the present. These results are consistent with our second hypothesis. Interestingly, Owen et al. (2013) found that both men and women who reported more thoughtful decision-making processes regarding their romantic relationship tended to report higher satisfaction with the relationship and fewer extradyadic behaviors.

Previous research has shown that individuals that have stronger sexual interest levels tended to have more extradyadic behaviors (Treas and Giesen, 2000). In addition, individuals who have betrayed in the past tend to report more unrestricted sociosexuality (Rodrigues et al., 2017). When we analyzed the association between having betrayed and the PRQC and SDR we found similar results. More specifically, individuals that had been betrayed by a partner tend to have higher sexual desire, and to perceive their romantic relationship has having lower quality. These results are consistent with our third hypothesis. These results may be explained due to the fact that those individuals that tend to betrayal also tend to be betrayed. These findings are consistent with Shaw et al. (2013) prospective study, that showed that partner’s extradyadic behaviors is a predictor of extradyadic relationships. More specifically, they found in a large, nationally representative sample of unmarried couples that factors such as lower relationship satisfaction, negative communication, and partner’s extradyadic behaviors (actual or suspected) were predictors of extradyadic sexual interaction. Research has shown that when men believe their partners are more likely to betray them, they feel more attracted to other women possibly to increase the likelihood of genetic transmission (Shaw et al., 2013).

Our data showed that there was no significant difference between those who had betrayed and had not betrayed regarding their self-perceived attractiveness. The same was true when we compared those who had been betrayed and had not been betrayed. These results are inconsistent with our second and third hypothesis. One possible explanation for these results is that because participants that have been betrayed are also more likely to have betrayed (Shaw et al., 2013), any possible differences were minimized.

Our results showed that, overall, those who perceive themselves as being more attractive tend to have a higher sexual desire and higher relationship quality. These confirm our fourth hypothesis. There are however, further gender differences. Specifically, women who perceived themselves as being relatively more attractive had a tendency to report a higher sexual desire than those who perceived themselves as being relatively less attractive. This result was not obtained for men. Previous research has shown that women who consider themselves physically attractive show a greater preference for masculinity and symmetry, suggesting that these women may attempt to maximize phenotypic quality in potential partners, whereas women of low mate value may maximize reproductive success by searching males most likely to invest (Little et al., 2001). Also, women (and not men) who perceived their relationship as high quality tended to give less importance to the partner’s physical attractiveness compared with those women who rated their relationship quality as low. This finding is consistent with an evolutionary perspective, suggesting that those women who are in a secure and committed relationship which provides good resources for themselves and the children are more likely to disregard physical attractiveness (Penton-Voak et al., 2003). Finally, men that perceived their relationship has having high quality were more likely to perceive themselves as more attractive.

### Limitations

First, even though we propose that past extradyadic behaviors history has an impact on the experience of current romantic relationships, our data were correlational and consequently we cannot make strong inferences. It is possible that someone with an overall high sexual desire and that tends to perceive the quality of intimate relationships to be low, will have a higher tendency to betray their partners during the course of their lives. Therefore, it would be very interesting to test which path is the most likely to occur by doing a prospective, longitudinal study. Second, we did not have an equal number of males and females’ participants that have betrayed/been betrayed. This unequal sample sizes may have affected the results (Keppel, 1993). Third, we did not ask participants about their perception of extradyadic behaviors, nor to specify the extradyadic behaviors them have suffered and/or inflicted. This may have affected the results. Fourth, we did not ask participants if they were in sexually non-monogamous relationships (SNMR), defined as those relationships in which “individuals are each other’s primary partners and have consensually agreed upon extradyadic sex” (Rodrigues et al., 2016). Research has shown that individuals in SNMR do not perceive extradyadic sex as a transgressive behavior or extradyadic behaviors (Mogilski et al., 2017). Therefore, having extradyadic sex does not seem to affect, for example, the quality of the relationships in SNMR (Mogilski et al., 2017), which may have affected our results. Fifth, research has shown that self-perceived attractiveness seems to be related with both face and body features, and that with ratings of attractiveness given by independent evaluators (Muñoz-Reyes et al., 2015). Nevertheless, it is possible that some participants may have confounded between face and body attractiveness. Sixth, all obtained correlation coefficients were low, and therefore strong inferences should not be done. Seventh, the age of the majority of our participants ranged from 18 to 40. It would be interesting to investigate if the same pattern of results would be obtained with an older sample. Finally, to evaluate some of our variables (e.g., extradyadic behaviors) we developed specific questions that have not been used in previous studies, which may have also affected our results.

## Conclusion

Overall, results of the present study add to our understanding of variables that play an important role in romantic relationship – perception of relationship quality, sexual desire, importance attributed to partner’s physical attractiveness, and self-perceived attractiveness – by showing there is a strong relationship between them and having betrayed a partner, or having been betrayed by a partner in the past. Results contributed to the literature by proving further evidence of gender differences, namely that men report higher levels of extradyadic behaviors than women, higher levels of sexual desire, attribute more importance to physical attractiveness and perceived their current relationship as having less quality. Results have implications for clinical settings. If we have a broader understanding of the variables that are related with extradyadic behaviors clinicians may be able to work more effectively with couples. More specifically, by working on those different variables – for example, by improving the relationship quality – clinicians will help reducing the probability of extradyadic behaviors and their negative consequences. Future research should use methodologies that would allow for stronger tests of the hypothesis that there is a causal linkage between extradyadic behaviors and later experience of other intimate relationships an individual may have.

## Data Availability Statement

The datasets generated for this study are available on request to the corresponding author.

## Ethics Statement

All procedures performed in studies involving human participants were in accordance with the ethical standards of the institutional research committee and with the 1964 Helsinki declaration and its later amendments or comparable ethical standards. Informed consent was obtained from all individual participants included in the study. This article does not contain any studies with animals performed by any of the authors.

## Author Contributions

JA designed the study. FB collected the data. JA and HO analyzed the data. All authors had important discussions. JA and FB wrote the first draft of the manuscript.

## Conflict of Interest

The authors declare that the research was conducted in the absence of any commercial or financial relationships that could be construed as a potential conflict of interest.

## Footnotes

1 www.qualtrics.com
